# Response of Wheat and Sugar Beet to Different Mineral–Organic Fertilization in a Long-Term Experiment

**DOI:** 10.3390/life15111779

**Published:** 2025-11-20

**Authors:** Przemysław Barłóg, Lukáš Hlisnikovský, Remigiusz Łukowiak, Ladislav Menšík, Eva Kunzová

**Affiliations:** 1 Department of Agricultural Chemistry and Environmental Biogeochemistry, Poznan University of Life Sciences, Wojska Polskiego 71F, 60-625 Poznan, Poland; remigiusz.lukowiak@up.poznan.pl; 2 Department of Nutrition Management, Crop Research Institute, Drnovská 507, Ruzyně, CZ-161 01 Prague 6, Czech Republic; lukas.hlisnikovsky@carc.cz (L.H.); ladislav.mensik@carc.cz (L.M.); eva.kunzova@carc.cz (E.K.)

**Keywords:** balanced fertilization, crop rotation, macronutrient uptake, nitrogen use efficiency, phosphorus, potassium, soil mineral nitrogen

## Abstract

The effect of cyclic pig slurry (PS) application in long-term crop rotations with alfalfa is poorly recognized, particularly with regard to nitrogen use efficiency (NUE) in crops requiring relatively high nitrogen (N) inputs. A long-term field experiment was established in Prague-Ruzyně, Czechia, in 1955. The experiment evaluated the effects of eight fertilization combinations, involving PS application and various N, phosphorus (P) and potassium (K) rates (N_0_P_0_K_0_; N_1_P_1_K_1_; N_3_P_2_K_2_; and N_4_P_2_K_2_). The effect of fertilization was evaluated in a 9-year crop rotation, in which PS was applied only three times under root crops. Long-term different mineral fertilization treatments and the application of PS significantly affected the yield of the tested crops: winter wheat and sugar beet. The highest wheat yield (8.34 t ha^−1^) was observed in the PS+N_3_P_2_K_2_ treatment, while the highest beet yield (86.1 t ha^−1^) was recorded in the PS+N_4_P_2_K_2_ treatment. The differences compared with the absolute control (N_0_P_0_K_0_) were 62.3% and 40.5%, respectively. However, statistically significant differences between treatments with different NPK rates were recorded only in plots without PS. With increasing NPK fertilizer rates, the uptake of macronutrients by plants also increased. The only exception was calcium in sugar beet in PS plots. The total N accumulation in plants was proportionally related to the total N input to the soil–plant system (N_in_). For winter wheat, this trend was beneficial, as it resulted in higher protein yield, whereas in beet, the sugar yield did not increase significantly when N_in_ exceeded 250 kg N ha^−1^. The obtained results indicate that, in the soil conditions of this experiment, N rates should be primarily balanced with appropriate rates of phosphorus.

## 1. Introduction

Long-term field experiments show that the best yield-forming effects are achieved under conditions of the simultaneous use of manure and mineral fertilizers [1,2,3,4,5]. There are many reasons for this phenomenon. Manure fertilizers, including pig slurry (PS), have a positive effect on soil organic matter (SOM), total nitrogen (TN) content, soil structure and compaction, water retention, cation exchange capacity (CEC), and the content of plant-available forms of nutrients [6,7,8,9,10]. Compared to various manures, PS is characterized by a relatively low concentration of dry matter (5–8%), narrow C:N ratio (3.6–4.6), and a high concentration of ammonium nitrogen (NH_4_-N), about 65–70% of total N content [11]. Due to their high NH_4_-N content, mineral N fertilizers can be partially or even completely replaced in fertilization systems [12]. In addition, the application of PS, a fertilizer with a low C:N ratio, can also cause a phenomenon known as the “priming effect”. As a result, plants will have a larger pool of mineral nitrogen (N_min_) available to them as a result of the mineralization of previously accumulated SOM in the soil [13]. Although the positive effect of PS application on plant N supply is well known [14,15], the long-term effect of fertilizer on mineral N (N_min_) accumulation in the soil is still poorly understood. This is especially true for long-term crop rotations where PS is not applied every year, but only cyclically for specific crops. The residual effect of N application in slurry, resulting from the mineralization of organic matter and the slow release of ammonium N immobilized in the soil, may last for several growing seasons [16]. In addition, the soil N_min_ content is subject to strong seasonal variations, which are due not only to the amount of fertilizer applied, but also to the microbiological activity, soil properties, and crops in the rotation [17,18,19].

In the context of crop rotation, the application of PS not every year but only before selected crops raises specific issues. If PS is applied only before one crop in the sequence, that crop can utilize the supplied N. In subsequent years of the rotation, before a crop that did not receive PS, either a positive “residual N effect” may occur or, conversely, an N deficiency if the plant’s N demand exceeds the supply from the soil and mineral fertilizers [20]. Depending on the structure of the crop rotation, an imbalance in N supply for successive crops may occur, which can lead either to the accumulation of soil N_min_ or to its loss from the soil [21]. An additional issue is the cultivation of leguminous plants in the rotation. These species are an important source of N_min_ in soil (through N_2_ fixation from atmosphere) and can reduce the need for high N rates in crop sequences [22]. However, the continuous inflow of N as a result of long-term PS application may reduce the potential of legumes to fix atmospheric N_2_ and, consequently, decrease the total N input to the soil [23]. As a result, the inclusion of legumes such as alfalfa in the rotation may mask differences in soil N_min_ content between plots fertilized and unfertilized with N. In addition to understanding the long-term effects of PS applications on soil N_min_, an important issue with both local and global significance is the improvement in nitrogen use efficiency (NUE) [24]. This indicator is a function not only of the N inputs in the soil–plant continuum but also of the plant’s supply of other nutrients [25]. In this context, the positive role of PS is evident, as it provides key macronutrients such as phosphorus (P) and potassium (K) [26,27]. Studies indicate that in rotations with PS, the rates of both these nutrients in fertilizers can also be reduced [4,6]. Nevertheless, it is always necessary to consider how the application of PS affects the content of plant-available forms of P and K in the soil, as these will determine the N uptake and NUE [28,29,30]. Therefore, another practical issue to address in long-term rotations with PS is the determination of the optimal N-to-PK ratio to maximize NUE. In other words, the research problem can be formulated as follows: does the cyclical application of pig slurry (PS) in a long-term crop rotation that includes alfalfa improve NUE indices in wheat and sugar beet—crops that take up relatively large amounts of N from the soil?

In the study, it was hypothesized that the regular application of PS in root crops in a 9-year crop rotation increases crop yield, macronutrient uptake, and improves the NUE in the soil–plant system. To verify this hypothesis, the results of a long-term experiment (65 years) were used to evaluate the effect of cyclic application of PS and different NPK rates on the following: (i) yield of winter wheat and sugar beet; (ii) accumulation of macronutrients in plants at harvest; (iii) indices of nitrogen use efficiency; and (iv) relationships between the content of macronutrients in the soil, especially N, and plant yield.

## 2. Materials and Methods

The results presented in this article come from a long-term Ruzyně Fertilizer Experiment established in the year 1955 in Prague-Ruzyně, Czechia (50.088236 N, 14.290417 E). The experiment uses a randomized complete block design. This experimental design involves five fields, each with 96 plots, where 24 different fertilizer treatments are replicated four times. The area of one experimental plot is 144 m^2^ (12 × 12 m). For the purposes of this study, Field Strip III was selected for analysis. It represents crop rotation referred to as ‘Classical Crop Rotation’. The proportions of each crop group are as follows: 45% cereals, 33% of root crops, and 22% of legumes. The crops are rotated in the following order: alfalfa–alfalfa–winter wheat–sugar beet–spring barley–potatoes–winter wheat–sugar beet–spring barley with alfalfa undersowing. This study evaluated the effect of long-term fertilization on the following two crops: winter wheat and sugar beet. For the purpose of this article, we evaluated 10 selected fertilization treatments as follows: N_0_P_0_K_0_; N_1_P_1_K_1_; N_3_P_2_K_2_; N_4_P_2_K_2_; PS+N_0_P_0_K_0_; PS+N_1_P_1_K_1_; PS+N_3_P_2_K_2_; and PS+N_4_P_2_K_2_, where PS denotes pig slurry application. Table 1 presents the rates of mineral fertilizers applied in 2020–2021 for winter wheat and sugar beet. In a 9-year crop rotation, PS is applied three times, only under root crops. The PS rates were 49 and 68 t ha^−1^ for potatoes and sugar beet, respectively. The basic parameters characterizing PS were as follows: pH—6.7; dry matter (DM)—42.3 g kg^−1^ of fresh matter; ash—166.8 g kg^−1^ of DM; total nitrogen—25.1 g kg^−1^ of DM; total phosphorus—9.8 g kg^−1^ of DM; total potassium—23.2 g kg^−1^ of DM. The total input of macronutrients to the experimental plots during the winter wheat and sugar beet seasons, supplied through mineral NPK fertilizers and pig slurry (PS), is presented in Table 1.

Within the current crop rotation system, PS was applied in 2016, 2018, and 2020, preceding the cultivation of sugar beet, potato, and sugar beet, respectively. The fertilizer was applied in autumn (October), following the harvest of the preceding cereal crops, barley, and wheat. Immediately after PS application, the soil was mixed with a medium harrow. Mineral P and K fertilizers (superphosphate: 8.3% P, and potassium chloride: 49.8% K) were also applied in autumn. Nitrogen was applied in the form of calcium ammonium nitrate (27% N). In wheat, a single dose was applied in early spring, before the start of vegetation. In sugar beets, N rates were applied twice: before sowing and at the 4-leaf stage. During the growing season, standard plant protection against weeds and pathogens was used.

According to the soil classification of the World Reference Base for Soil Resources [31], the soil type at the study site is Orthic Luvisol. In terms of particle-size fraction composition, the soil is classified as silty clay loam. Basic soil parameters, along with the content of plant-available forms of nutrients, are presented in Table 2. The content of nutrients in the soil depended on the fertilization treatment, hence, the results are given in ranges.

### 2.1. Weather Conditions

**Figure 1:**
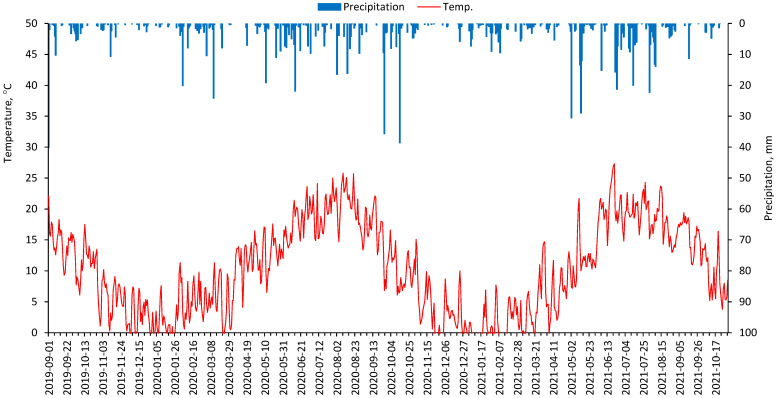
Mean daily air temperature and precipitation during growing seasons of winter wheat (2019–2020) and sugar beet (2021). Data from the meteorological station in Prague-Ruzyně.

The average long-term (1955–2019) temperature in the study area is 8.2 °C (ranging from 6.4 to 9.7 °C) and the annual sum of precipitation is about 450 mm (ranging from 255 to 701 mm). In 2020–2021, the average temperature was 10.6 and 9.2 °C. Total precipitation was 505 and 530 mm, respectively. Detailed meteorological data from autumn 2019 (wheat sowing) to autumn 2021 (sugar beet harvest) are provided in Figure 1. In the 2019/2020 winter season, average temperatures and precipitation were higher than in 2020/2021 winter. In April, precipitation was low in both growing seasons; however, this did not have a major impact on plant growth because in March, regardless of the year of the study, precipitation was sufficient to meet the water needs of the plants. In May and June, precipitation was insufficient to varying degrees, especially in 2021. In the summer of 2021, precipitation was also high in July and August, which are critical months for sugar beet yields. Only in September and October 2021 did the total precipitation decrease and fall below the long-term average. This weather pattern had a negative impact on leaf growth in the final stage of sugar beet growth.

### 2.2. Soil Material and Chemical Analysis

Soil samples were collected in early spring, before the start of vegetation, in the following two growing seasons: 2019/2020 and 2020/2021. Soil samples were taken from two layers: topsoil (0.0–0.3 m) and subsoil (0.3–0.6 m). The soil sample representing one plot was made by mixing several individual samples taken from the same soil layer. NH_4_-N and NO_3_-N were determined in field-fresh soil samples. Twenty-grams of soil samples were shaken for 1 h with 100 mL of a 0.01 M CaCl_2_ solution [17]. Concentrations of NH_4_-N and NO_3_-N were determined with the colorimetric method using flow injection analyses (FIAstar 5000, FOSS, Hillerød, Denmark). The concentration analysis of NO_3_-N consisted of two basic steps: reduction from nitrate to nitrite (NO_2_-N), and then the reaction of nitrites with N-(1-naphtyl)ethylene-diamine dichloride as a diazotizing agent (Griess-Ilosvay reaction). The measurement was performed at a wavelength of 540 nm. To determine NH_4_-N, a special ammonia indicator (mixture of cresol red, bromocresol purple, and bromothymol blue) was applied. The measurement was made at a wavelength of 590 nm. The soil mineral nitrogen (N_min_) was calculated as the sum of NH_4_-N and NO_3_-N, expressed in kg ha^−1^.

Soil samples were dried at room temperature (~20 °C) for the remaining chemical determinations. Next, the soil samples were ground in a porcelain mortar and then sieved to obtain fractions smaller than 2 mm. The content of plant-available micronutrients (P, K, Mg, and Ca) was determined by the Mehlich 3 method [32]. The concentration of P in the Mehlich 3 extract was measured by the ammonium molybdate method using a Jasco V-630 UV-VIS spectrophotometer (Jasco International Co., Ltd., Tokyo, Japan) at a wavelength of 800 nm. The concentrations of cations (K, Mg, and Ca) in the Mehlich 3 extract were analyzed by atomic absorption spectrometry 235 (AAS) (ThermoScientific iCE 3000 Series, Thermo Fisher Scientific Inc., Waltham, CA, USA).

### 2.3. Plant Material and Chemical Analysis

Winter wheat (*Triticum aestivum* L.) of the variety Mulan was tested in the first year and sugar beet (*Beta vulgaris* L.; variety BTS 6995) in the second year. Winter wheat was harvested on 8 August 2020, and sugar beet on 4 October 2021. Wheat was harvested with a ‘Sampo’ combine harvester from an area of 25 m^2^. Grain yield (GY) and straw yield (SY) of each plot were recalculated to t ha^−1^ at 85% dry matter. Sugar beet was harvested manually from an area of 10 m^2^. From each plot, samples of wheat grain and straw, and beet taproot and leaves were randomly selected for chemical analysis. After drying and grinding, the N content was determined by the Kjeldahl method using a thermal block and a distillation unit (FOSS, Denmark). In order to determine the remaining macronutrients, plant material was mineralized using the dry ashing method at a temperature of 600 °C. The concentration of P, after dissolving the ashes in dilute HNO_3_, was measured by the colorimetric method with ammonium molybdate using a Jasco V-630 UV-VIS spectrophotometer (Jasco International Co., Ltd., Tokyo, Japan) at a wavelength of 436 nm. The concentration of K, Mg, and Ca were measured using atomic absorption spectrometry (ThermoScientific iCE 3000 Series, Thermo Fisher Scientific Inc., Waltham, CA, USA). The total uptake of macronutrients at the final growth stage was computed as the sum of nutrient uptake in crop yield (grain or taproots) and harvest residues (straw and leaves). The harvest index (HI) is the ratio of the amount of the nutrients accumulated in the main yield to their total accumulation.

### 2.4. Nitrogen Use Efficiency Indices

In order to assess the nitrogen use efficiency (NUE) and N balance in the soil–plant system, the following indices were used [33]:

Partial factor productivity of N_f_ (PFP) = Y/N_f_ [kg Y kg^−1^ N_f_] (1)

Agronomic efficiency of N_f_ (AE) = (Y − Y_0_)/N_f_ [kg Y kg^−1^ N_f_] (2)

Partial N_f_ balance (PNB) = TN/N_f_ [kg N_t_ kg^−1^ N_f_] (3)

N utilization efficiency (NUtE) = Y/TN [kg Y kg^−1^ N_t_] (4)

Biomass productivity of N_in_ (NUEs) = (Y + R)/(N_min_ + N_f_) [kg kg^−1^ N_in_] (5)

Partial factor productivity of N_in_ (PFP_in_) = Y/(N_min_ + N_f_) [kg Y kg^−1^ N_in_] (6)

N uptake efficiency (NUpE) = N_t_/(N_min_ + N_f_) [kg N_t_ kg^−1^ N_in_] (7)

N input/output balance (NUEb) = YN/N_f_ [kg N ha^−1^] (8)

where Y—wheat grain yield or sugar beet taproot yield, kg ha^−1^; Y_0_—wheat grain or sugar beet taproot yield in treatment without N fertilizers, kg ha^−1^; N_f_—total N input in fertilizers, kg N ha^−1^; N_t—_total N accumulation in above-ground biomass of wheat and sugar beet, kg N ha^−1^; R—crop harvest residues, kg ha^−1^; N_in_—total N input from the soil (N_min_, 0.0–0.60 m) and mineral fertilizers (N_f_), kg N ha^−1^; YN—N in wheat grain or sugar beet taproots, kg N ha^−1^.

### 2.5. Statistical Analysis

The effect of fertilizer treatments on soil macronutrient content, crop yield, nutrient uptake, and NUE indices was evaluated using one-way ANOVA. Means were separated by honest significant difference (HSD) using Tukey’s method. The distribution of the data (normality) was checked by the Shapiro–Wilk [34] test and the homogeneity of variance by the Bartlett test. The standard error of the mean (SEM) was used to express the statistical error. The relationships between traits were analyzed using Pearson’s correlation and nonlinear regression. Statistica 13.3 software was used for all statistical analyses (TIBCO Software Inc., Palo Alto, CA, USA).

## 3. Results

### 3.1. Crop Yield

**Figure 2:**
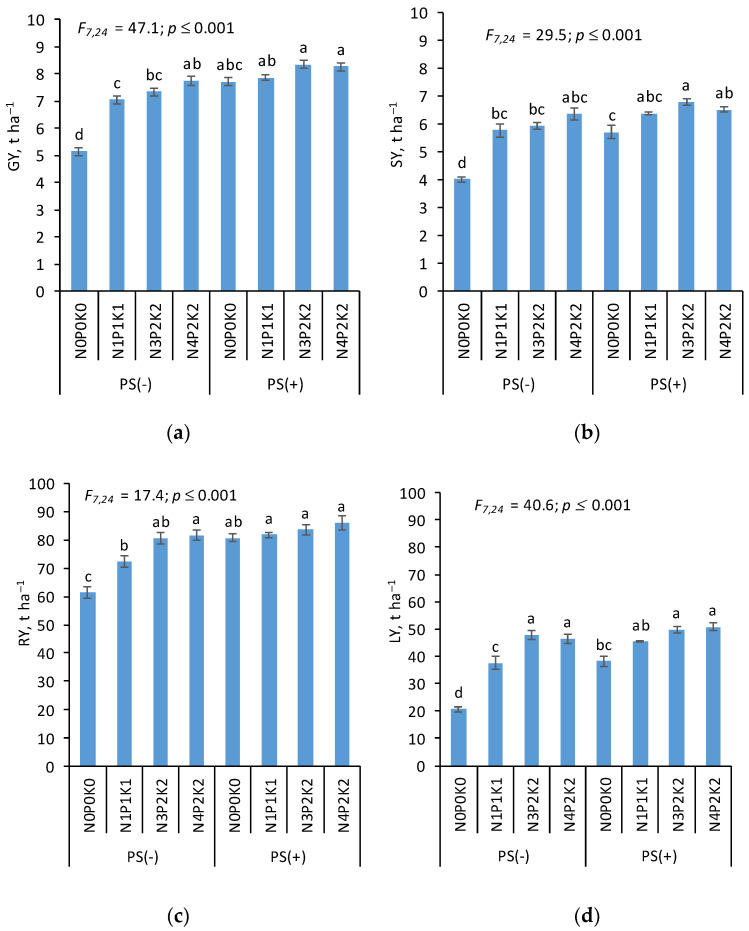
Yield of grain (**a**) and straw (**b**) of winter wheat and taproots (**c**) and leaves (**d**) of sugar beet. Key: PS(−) plots without pig slurry; PS(+) plots with pig slurry in the crop rotation. Detailed description of NPK doses for each treatment is given in Table 1. Different letters indicate significant differences between treatments (*p* ≤ 0.05). Hatched bars represent the 2 ± SEM ranges.

Long-term application of different NPK rates significantly affected crop yield and crop residue biomass (Figure 2). In the plots without PS, the highest yield of grain (GY) and straw (SY) of winter wheat was obtained in the N_4_P_2_K_2_ treatment. These values were 7.74 and 6.35 t ha^−1^, respectively. Compared to the control (N_0_P_0_K_0_), the increase in GY and SY was 50.1% and 58.8%, respectively. In the PS plots, the highest GY and SY were obtained in the treatment with a moderate N rate (PS+N_3_P_2_K_2_). The GY and SY values in this treatment were 8.35 and 6.78 t ha^−1^, respectively. The difference between the control and the PS+N_3_P_2_K_2_ treatment was 62.3% for GY and 69.5% for SY. Regardless of NPK fertilizers rates, the difference in GY and SY between the PS(−) and PS(+) treatments was 18.6% and 14.9%, respectively (Figure 2a and Figure 2b).

In relation to sugar beet, the highest taproot yield (TY) was observed in the treatment with the highest N rate (N_4_), regardless of PS application. However, in the fertilization system without PS, the TY increase compared to the control was lower (+33.1%) than with PS (+40.5%). At the same time, application of PS alone increased TY by 31.5% compared to the control. The effect of PS on TY was therefore comparable to that of mineral-fertilizer-only treatments with a medium N rate (N_3_P_2_K_2_). The greatest increase in LY was also obtained in the PS+N_4_P_2_K_2_ treatment. In this treatment, LY was 2.5 times higher than in the control. On average, the application of PS increased TY by 12.2% and LY by 19.0% compared to the values obtained in the plots without PS (Figure 2c,d).

The study found that mineral fertilization significantly increased the main yield (GY and TY) of the tested plants in the absence of PS. Under conditions with PS application, the biomass of crop residues changed more considerably than the main yield.

### 3.2. Macronutrient Uptake

Fertilization had a significant effect on the total uptake of all macronutrients by the tested plants. Based on the coefficient of variation (CV, %) as an indicator of the fertilization effect on the trait studied in wheat, the macronutrients can be ranked as follows: K_t_ (12.8) < P_t_ (13.9) < Mg_t_ (15.4) < Ca_t_ (19.0) < N_t_ (19.1). In general, the accumulation of macronutrients increased with higher levels of NPK fertilization (Table 3).

The highest uptake of N_t_, P_t_, Ca_t_, and Mg_t_ was recorded in the PS+N_4_P_2_K_2_ treatment. Only for K_t_ was a higher value observed in the N_4_P_2_K_2_ treatment compared to PS+N_4_P_2_K_2_. The application of PS enhanced total macronutrient accumulation regardless of the level of mineral fertilization. On average, the differences in macronutrient accumulation between plots without and with PS were as follows: N_t_—25.2%; P_t_—14.9%; K_t_—6.1%; Ca_t_—15.2%; and Mg_t_—22.5%. For comparison, the differences in uptake between the absolute control (N_0_P_0_K_0_) and the PS-only treatment (PS+N_0_P_0_K_0_) were even greater as follows: 48.8%, 38.7%, 36.3%, 29.4%, and 40.1% for N_t_, P_t_, K_t_, Ca_t_, and Mg_t_, respectively. The study also found that mineral fertilization (N_4_P_2_K_2_) increased total macronutrient accumulation in wheat to a greater extent in plots without PS (N_t_—78.2%; P_t_—56.6%; K_t_—64.3%; Ca_t_—62.1%; Mg_t_—52.1%) than in those with PS (N_t_—36.4%; P_t_—21.5%; K_t_—15.2%; Ca_t_—57.2%; Mg_t_—19.5%).

For sugar beet, the highest coefficient of variation (CV) was also recorded for N_t_ (27.4%). Fertilization had a strong influence on the accumulation of P_t_ and K_t_ (CV = 21.1% and 19.4%, respectively), while its effect on Ca_t_ and Mg_t_ was the weakest (CV = 11.1% and 13.7%). The application of PS increased the average accumulation of N_t_, P_t_, K_t_, and Mg_t_ by 34.4%, 27.7%, 11.6%, and 14.7%, respectively. Only Ca_t_ levels remained comparable across treatments. On plots without PS, the highest dose of NPK mineral fertilizers significantly increased the accumulation of macronutrients compared to the control. The increases were 82.6% for N_t_, 96.0% for P_t_, 96.2% for K_t_, 37.8% for Ca_t_, and 53.1% for Mg_t_. In plots with PS, the highest NPK dose enhanced the accumulation of N_t_, P_t_, K_t_, and Mg_t_ by 89.5%, 29.7%, 41.1%, and 16.7%, respectively, but did not increase Ca_t_ uptake. In fact, for calcium, a decreasing trend was observed with increasing NPK rates. Consequently, the highest Ca_t_ uptake was recorded in the N_3_P_2_K_2_ and PS+N_1_P_1_K_1_ treatments.

Fertilization significantly affected the N and Ca accumulation index in wheat, as well as all macronutrients in sugar beet (Table 4). In wheat, NHI and CaHI values decreased with increasing NPK mineral fertilization levels. This trend was particularly pronounced in the PS-treated plots. The lowest CaHI index values were obtained in the PS+N_3_P_2_K_2_ treatment. As the total NPK dose increased, the nutrient accumulation index values in sugar beet roots also decreased. This trend, as with wheat, was mainly observed in the PS-treated plots. PS application alone caused a slight increase in NHI and a slight decrease in PHI compared to the absolute control. PS application significantly reduced relative Ca accumulation in roots.

In summary, total macronutrient uptake by plants increased with higher NPK rates, particularly in plots without PS. The exception was the accumulation of Ca_t_ in sugar beet plants.

### 3.3. Soil Mineral N

The tested soils were characterized by a significantly higher content of nitrate nitrogen compared to ammonium nitrogen. The average NH_4_-N content ranged from 2.72 to 5.34 kg ha^−1^, depending on the year and soil layer. The average NO_3_-N content varied from 44.4 to 57.9 kg ha^−1^ in the 0.0–0.3 m layer and from 27.4 to 39.1 kg ha^−1^ in the 0.3–0.6 m layer, depending on the year. In 2021, the fertilization treatments significantly affected NO_3_-N content, and consequently, the total mineral nitrogen (N_min_) content (Table 5). A clear relationship was observed as follows: the higher the nitrogen input from fertilizers, the greater the NO_3_-N concentration in the soil. Although no statistically significant differences were observed in 2020, a noticeable trend toward increased NO_3_-N content following NPK application was still evident. In the 0–0.3 m layer, the highest NO_3_-N content was recorded in the PS+N_4_P_2_K_2_ treatment. Similarly, in the 0.3–0.6 m layer, the PS+N_4_P_2_K_2_ treatment also resulted in the highest NO_3_^−^-N levels. This pattern was consistent across growing seasons. In 2021, high NO_3_^−^-N content was also observed in the PS+N3P2K2 treatment.

The influence of fertilization treatments on total N_min_ content across the two soil layers is presented in Table 5. In both years, N_min_ content increased with the applied NPK dose. However, only in the second year (2021) was a significant effect of fertilization on the total N_min_ content observed. The application of PS alone also increased N_min_ levels compared to the absolute control, regardless of the year of research.

### 3.4. Crop Yield and Nutrient Uptake as a Function of Soil Properties

The tested plants responded specifically to the soil’s chemical composition, particularly to the levels of essential macronutrients. In wheat, significant correlation coefficients were observed between grain yield (GY) and plant-available phosphorus (M3P) content, regardless of soil depth. In the case of potassium (M3K), significant correlations were found only in the 0.3–0.6 m layer (Table 6). Sugar beet also showed a positive response to soil M3P and M3K levels. Unlike wheat, stronger correlations were observed between total yield (TY) and mineral nitrogen (N_min_) content in both soil layers, as well as with M3K in the 0.0–0.3 m layer (Table 7). However, the nature of the relationships between traits was more complex and nonlinear (Figures S1 and S2). The relationship between GY and M3P content was best described by a second-order function, with the soil layer having no significant effect on the coefficient of determination:

0–0.3 m: GY = −0.0021M3P^2^ + 0.2986M3P − 2.2101; *R*^2^ = 0.89; *p* ≤ 0.05 (9)

0.3–0.6 m: GY = −0.0002M3P^2^ + 0.0642M3P + 2.2336; *R*^2^ = 0.90; *p* ≤ 0.05 (10)

The best fit of GY and M3K was found for the soil layer 0.3–0.6 m:

GY = −0.0024M3K^2^ + 0.8832M3K − 73.653; *R*^2^ = 0.72; *p* ≤ 0.05. (11)

Sugar beet yield was best explained by the content of M3P in the arable layer (0–0.3 m) and M3K in the layer 0.3–0.6 m:

TY = −0.0023M3P^2^ + 0.6037M3P + 46.185; *R*^2^ = 0.86; *p* ≤ 0.05 (12)

TY = −0.0227M3K^2^ + 8.9784M3K − 803.97; *R*^2^ = 0.70; *p* ≤ 0.05. (13)

The polynomial function was even more suitable for describing the relationship between soil mineral nitrogen (N_min_) and grain yield (GY) (*R*^2^ = 0.81, *p* ≤ 0.05 and *R*^2^ = 0.73, *p* ≤ 0.05 for the 0.0–0.3 m and 0.3–0.6 m soil depths, respectively).

**Figure 3:**
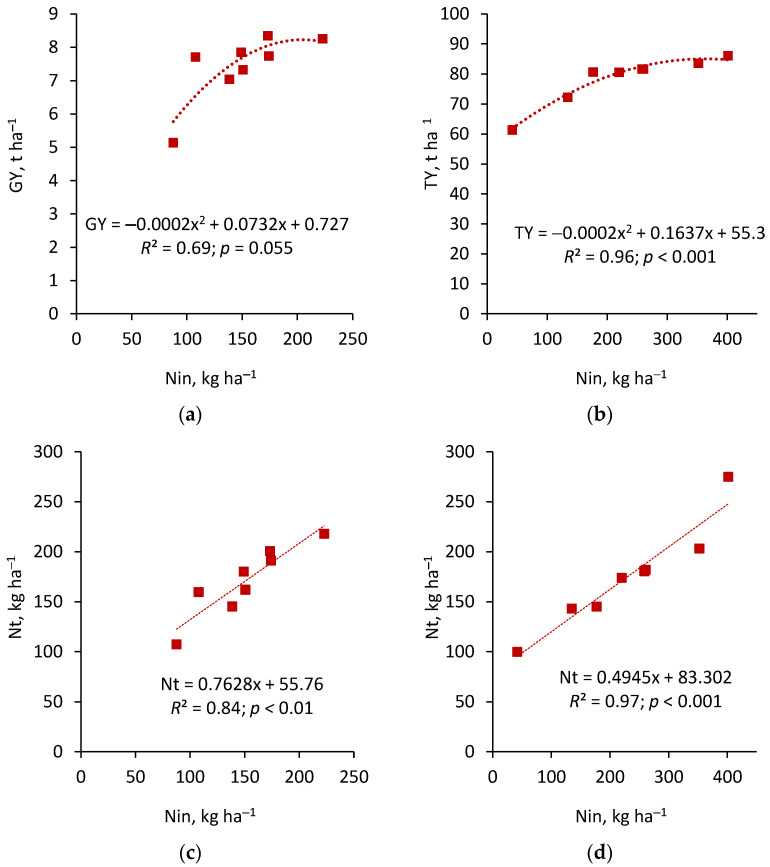

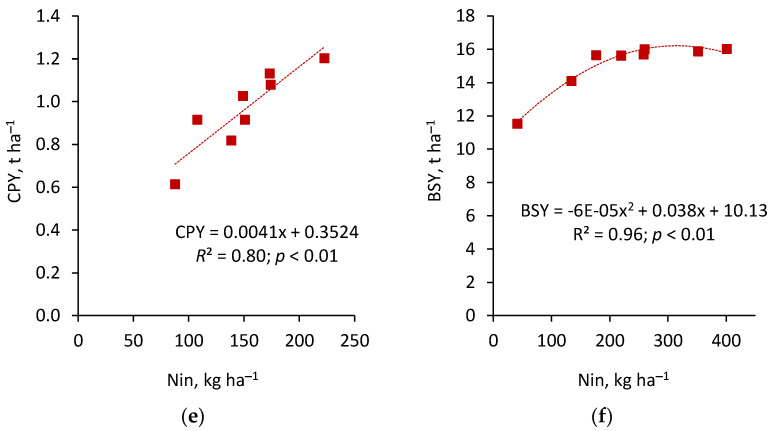
Winter wheat grain yield, GY (**a**); sugar beet taproot yield, TY (**b**); total N uptake (Nup) by wheat (**c**) and sugar beet (**d**); wheat crude protein yield, CPY (**e**); and biological sugar yield of sugar beet, BSY (**f**) as a function of spring N input (Nin) to the soil–plant system.

Mineral fertilizer applied in spring, after soil sampling, also served as a source of nitrogen for the plants. Therefore, analyzing the relationship between GY and the total N input into the soil–plant system (N_in_) is important. As shown, GY increased significantly with N_in_ up to approximately 190 kg ha^−1^ (Figure 3a). Total nitrogen (N_t_) uptake, as well as crude protein yield in wheat (CPY) plants, increased proportionally with N_in_ into the soil–plant system (Figure 3c,e). Unlike wheat, for sugar beet, the greater the spring nitrogen input to the soil–plant system, the higher the taproot yield (TY). The relationship between TY and N_in_ was best described by a second-degree polynomial function, while the relationship between total nitrogen uptake (N_t_) and N_in_ was best explained by a linear function (Figure 3d). It is worth emphasizing that the biological sugar yield (BSY) increased only to a certain level, corresponding to N_in_ at the level 250 kg ha^−1^ (Figure 3f).

In summary, unbalanced N rates negatively affect the efficiency of N utilization accumulated in plants.

### 3.5. Nitrogen Use Efficiency

The tested fertilization variants significantly affected the NUE indices. In general, with increasing N fertilizer rates, NUE indices decreased on both PS(−) and PS(+) plots. In wheat cultivation, the highest values of classic fertilizer-based indices (PFP, AE, PNB) were obtained at the N_1_P_1_K_1_ mineral fertilization level. At each mineral fertilization level, these indices were higher on plots cyclically fertilized with PS. As a result, the average PFP, AE, and PNB values on PS(+) plots were 11.1%, 37.7%, and 32.9% higher, respectively, than on PS(−) plots. Positive PNB values indicate soil mining, which was more pronounced on PS(+) plots. The NUtE, NUEs, PFPin, and NUpE indices also decreased with increasing soil N input, but the highest values for these indices were observed in the PS+N_0_P_0_K_0_ treatment, not in N_0_P_0_K_0_. The first index (NUtE) reflects the yield-forming role of N accumulated in the plant, while the latter three emphasize the efficiency of total N input in spring (mineral fertilizers and soil) for yield formation. Regardless of the mineral fertilization level, PS application led to a 6.2% reduction in the productivity of N accumulated in the plant (NUtE). For the other indices (NUEs, PFPin, and NUpE), values increased from 91.3, 50.6, and 111.2 kg kg^−1^ to 92.9, 52.1, and 120.0 kg kg^−1^, respectively. Positive NUEb values indicate wheat’s use of soil N resources, especially on PS(+) plots. The decreasing index values with increasing NPK rates indicate lower soil mining (Table 7).

For sugar beet, PFP values in the N_1_P_1_K_1_ and PS+N_0_P_0_K_0_ treatments were at similar levels. On average, however, PFP in mineral fertilizer treatments was lower on PS-treated plots compared to non-PS plots. The AE and PNB indices were highest in the PS+N_0_P_0_K_0_ treatment, but simultaneous application of PS and NPK fertilizers reduced AE and PNB values. The NUtE, NUEs, PFPin, and NUpE indices decreased with soil N input. Unlike wheat, the highest index values were observed in the control treatment (N_0_P_0_K_0_). On average, these indices were lower in PS(+) treatments than in PS(−) treatments. However, it is worth noting that at the N_4_ treatment level, PS application significantly improved the NUpE value. The NUEb index decreased with increasing N rate, but unlike wheat, the values were less than 1, indicating N accumulation in the soil. Only in the PS+N_0_P_0_K_0_ treatment was the index equal to 1.0, indicating a balance between N input and output from the soil–plant system (Table 7).

## 4. Discussion

### 4.1. Soil Proporties

Numerous research results indicate that the application of PS modifies the chemical properties of soils [27]. However, the degree of the reaction depends on a number of factors, including soil properties, application rates, and frequency of PS application [4,7,9,35,36,37]. The effect of cyclic fertilization PS on the content of P and K in the studied long-term field experiment has already been described earlier [38]. The present study focuses on the analysis of N_min_ content. In this study, the highest content of N_min_ was found in spring 2020, not in 2021 after the autumn PS application. The reason was the higher temperatures during the winter in the first season with wheat, which consequently led to a higher rate of organic N mineralization [39]. Regardless of the season, with increasing N input to the soil from both mineral fertilizers and PS, the content of N_min_ also increased. In the case of treatments without PS but with mineral N fertilization only, this is possible because the overall return of organic N to the soil through roots, root exudates, and crop residues increases [40]. In this study, the greatest changes in soil N_min_ content were observed under conditions of long-term application of mineral N and simultaneous application of PS in autumn 2020. The latter fertilizer is a source of ammonium ions (NH_4_^+^) in the soil [11]. These ions can be biologically immobilized by microorganisms or strongly adsorbed by clay minerals, thereby increasing the total N pool in the soil [41]. As a result, as shown by this research, long-term application of PS improves the soil’s potential to supply plants with N, although the extent of the response depends on the soil type [42]. There is also a risk of NH_3_ emissions during its application; however, under the experimental conditions (acidic soils and good PS incorporation into the soil), the probable NH_3_ losses were minimal [16]. As demonstrated in the experiment, the main pathway of ammonium N transformation from PS was nitrification [43]. As a result of this process, nitrates accumulated in the soil in early spring, especially in 2021 following the autumn application of PS. A similar positive effect of autumn PS application on spring NO_3_-N content was reported by other authors [44]. Unfortunately, this form of N may be leached from the soil, particularly in fields with late-sown crops, such as sugar beet.

It should also be emphasized that alfalfa was included in the evaluated crop rotation. This plant is a significant source of N in the rotation, as through symbiosis with root nodule bacteria it can fix from 50 to 450 kg N ha^−1^ [45]. The net soil N balance indicated that an average of 84, 148, and 137 kg N ha^−1^ was added to the soil system for 1-, 2-, and 3-year-old alfalfa stands [46]. In the present study, the effect of alfalfa on N_min_ content was probably smaller, as the soil samples were analyzed in the fifth and sixth year after its cultivation. It is also worth mentioning that the increasing level of N_min_ in the soil as a result of N fertilization, including the application of PS, may reduce nodule formation and N_2_ fixation [47].

Pig slurry is also a source of macronutrients in the soil. Its long-term application generally increases the soil content of P and K; however, the extent of this effect depends on many factors [27]. Our previous studies have shown that soil samples collected in spring 2020 from the PS+N_4_P_2_K_2_ treatment contained less M3P in the topsoil than from the PS+N_3_P_2_K_2_ treatment [38]. A similar trend was found in 2021 for M3K, but in the 0.3–0.6 m range. These results indicate a slow depletion (or no accumulation) of plant-available P and K in soils under conditions of unbalanced N rates. It is important to emphasize that even the application of PS in crop rotation did not compensate for the losses of P and K from the soil caused by 65 years of applying the highest rates of N [38]. Over the long term, such a trend may result in a significant decline in crop yields. According to Chen et al. [48], imbalanced fertilization without K can, even on initially K-rich soils, lead to gradual depletion of K and reduction in crop yields. Regarding P, it is also important that N application may indirectly affect M3P content through increased soil acidification and formation of less plant-available forms of P [49].

### 4.2. Crop Yield and Nutrient Uptake

The average wheat grain yield in the Czech Republic between 2019 and 2023 ranged from 5.73 to 6.42 t ha^−1^, while the sugar beet root yield ranged from 61.5 to 69.6 t ha^−1^ [50] This yield level for both crops was lower compared to the values obtained in the long-term experiment. Only in the control variant, without fertilization, were the yields of both crops below the national average. Nevertheless, these yields were still relatively high considering 65 years without the application of mineral fertilizers and PS to the soil. One of the reasons may be the presence of alfalfa in the crop rotation, which enhances the overall soil fertility and compensates for the removal of nutrients with the harvested crops [51]. High wheat and sugar beet yields compared to the national average can also be explained by the fertilization level. The average use of NPK fertilizers in the Czech Republic in recent years amounted to 126.8, 18.2, and 9.6 kg ha^−1^ of N, P_2_O_5_, and K_2_O, respectively [50]. This corresponds to an N:P:K ratio of approximately 1:0.14:0.08. In the conducted experiment, nitrogen was better balanced, with N:P:K ratios depending on the fertilization treatment ranging from 1:0.35:1.33 to 1:0.53:2.0 for wheat, and from 1:0.26:0.88 to 1:0.35:1.56 for sugar beet. Numerous studies indicate that good balancing of N with other macronutrients improves the yield potential of plants [52,53]. Moreover, it stabilizes yields over the years, which is crucial in the face of climate change [54].

In the study, the highest wheat yield (GY) was obtained in the PS+N_3_P_2_K_2_ treatment, and the highest sugar beet yield in the PS+N_4_P_2_K_2_ treatment. The N:P:K ratio in fertilizer doses, as well as in PS, was 1:0.47:1.82 for wheat and 1:0.26:0.88 for sugar beets for these treatments. In the research presented, the highest N rate in mineral fertilizers (N_4_) had no significant yield-forming effect compared to N_3_ under the conditions of cyclic PS application. In wheat, even a slight reduction in GY was observed in the PS+N_4_P_2_K_2_ treatment compared to the PS+N_3_P_2_K_2_ treatment. Nitrogen is a critical macronutrient in agriculture, essential for plant growth, yield, and crop quality [55,56]. However, its utilization by crop plants depends on other nutrients, particularly P and K. Phosphorus play a key role in photosynthesis, signal transduction, biosynthesis of proteins, carbohydrates, and lipids, early root growth, response to abiotic stresses, and seed formation [57]. Potassium is involved in CO_2_ assimilation, plant growth, nitrogen compound conversion, biosynthesis of proteins, lipids, and other compounds, as well as tolerance to water stress [58]. Therefore, too high an inflow of N in relation to P and K in fertilizers was the reason for the poor utilization of the N_4_ rate on plots cyclically fertilized with PS. This hypothesis is supported by earlier studies [2,4,5,29]. An analysis of the long-term experiment in Rothamsted (United Kingdom) showed that long-term application of N alone, without proper nutrient balance and management of P and K, may limit the achievement of crop yield potential [40].

Regardless of the growing season, N accumulation in plants (N_t_) showed the greatest variability, clearly indicating that the main factor responsible for this result was the varying N inputs (N_in_) in the crop rotation. The primary factors influencing N_in_ were the N rate from mineral fertilizers and PS. Moreover, in the PS treatment, the contribution of N_min_ accumulated in the soil due to the so-called residual effect of PS fertilization was also significant [59,60]. In general, N_t_ increased proportionally with the increase in N_in_. In the wheat season, the yield-forming response to N_in_ in the soil–plant system was nonlinear. The mathematical model determined the critical N_in_ value at about 190 kg ha^−1^. It is worth noting, however, that although grain yield did not increase significantly, the higher N supply had a positive effect on grain quality (Table S1), and the total protein yield was highest in the PS+N_4_P_2_K_2_ treatment. In relation to sugar beet, excessive N_in_ led to a decrease in the quality of the taproots-sucrose content (Table S1), and thus no significant increase in biological sugar yield above 250 kg ha^−1^. This result highlights the challenge of converting accumulated N_t_ into yield under conditions of unbalanced N application rates. The N rate imbalance was also reflected in the ratio of NPK accumulation in wheat plants: N_t_ increased up to N_4_, but P_t_ and K_t_ did not increase significantly compared to N_3_, regardless of the PS treatment.

### 4.3. Nitrogen Use Efficiency

Several nitrogen use efficiency (NUE) indices, often considered fertilizer-based indices (e.g., agronomic efficiency, AE), show low NUE values in long-term experiments [33]. Nevertheless, some authors indicate that slurry application improves NUE (AE) values [29,61]. According to Hlisnikovsky et al. [54], however, including PS in mineral NPK fertilization significantly reduces NUE (AE). In our studies, the effects of PS application on NUE indices should mainly be considered through modifications of soil properties. In wheat, slurry was not applied; however, on plots with PS, significantly higher values of PFP, AE, and PNB indices were observed than on plots without PS. One possible explanation is the improved supply of P and K to plants under PS application [62]. Furthermore, the residual N from previous slurry applications lasts for three years. Thanks to this, it was possible to increase some NUE indices in crop rotation with mineral fertilizers by over 33% [63]. Thus, as confirmed by our own research, improving N_min_ content through PS application, even if not applied every year but only to selected crops in rotation, enhances N conversion into yield. Also, N accumulated in plants (PNB index) is better converted into yield under cyclic PS application conditions. This is related to the broad spectrum of positive effects of PS on soil properties, including the content of plant-available forms of P and K [38,64].

In this study, to increase the reliability of NUE assessment, indices were also used to evaluate the combined effect of N from soil and mineral fertilizers. The results obtained also confirm the positive effect of PS on NUE indices, especially in treatments where PS was the only N source for plants. However, as shown in the wheat season, the lowest NUpE value was obtained in the treatment with the highest N_min_ in the soil (PS+N_4_P_2_K_2_). The greatest risk of N losses to the environment was thus identified in this treatment. In sugar beet, NUpE values on fertilized plots were lower than for wheat. Furthermore, a gradual decrease in NUpE values was noted with increasing N rates, indicating lower N uptake from the soil. The directions of changes in N use from the soil in the cultivation of both crops are confirmed by the NUEb index. For wheat, values of the index > 1.0 indicate depletion of the nutrient from the soil, while for sugar beet, values < 1.0 indicate N accumulation in the soil [33]. The hypothesis of N accumulation in the soil is supported by the fact that with increasing N rates, spring N_min_ in the soil increases. This may be the effect of both accumulation of N not taken up by plants from fertilizers and greater N input from crop residues.

## 5. Conclusions

Long-term combined mineral and organic fertilization had a significant effect on the yield of selected crops, macronutrient uptake, and nitrogen use efficiency (NUE). In general, cyclically application of PS enhanced both wheat and sugar beet yield. However, on plots not fertilized with PS, the crop response to mineral fertilization was significantly higher than on plots regularly amended with PS. Continuous PS application increased the mineral N content in the soil (N_min_) and the N input to the soil–plant system, while simultaneously stimulating N uptake by plants. Nevertheless, the yields of wheat and sugar beet were more strongly correlated with plant-available soil P than with N_min_. Furthermore, in treatments with the highest N input, the productivity of N accumulated in the plant decreased, particularly in sugar beet. Under the conditions of the present long-term experiment, the optimal treatment was the combined application of PS and moderate N rates, well balanced with phosphorus (PS+N_1_P_1_K_1_ and PS+N_3_P_2_K_2_). The study clearly demonstrates that NUE indices can be improved by adjusting N application rates according to soil N_min_ content and by maintaining an appropriate balance between N and other macronutrients.

## Figures and Tables

**Table 1:** Total macronutrient (NPK) input to the experimental plots during the winter wheat and sugar beet seasons from mineral fertilizers and pig slurry (kg ha^−1^ year^−1^).

Treatments	Winter Wheat (2019–2020)			Sugar Beet (2020–2021)		
	N	P	K	N	P	K
N_0_P_0_K_0_	0	0	0	0	0	0
N_1_P_1_K_1_	40	21	80	80	28	125
N_3_P_2_K_2_	55	26	100	160	35	166
N_4_P_2_K_2_	75	26	100	200	35	166
PS+N_0_P_0_K_0_	0	0	0	89	39	88
PS+N_1_P_1_K_1_	40	21	80	169 (80 + 89)	67 (28 + 39)	213 (125 + 88)
PS+N_3_P_2_K_2_	55	26	100	249 (160 + 89)	74 (35 + 39)	254 (166 + 88)
PS+N_4_P_2_K_2_	75	26	100	289 (200 + 89)	74 (35 + 39)	254 (166 + 88)

The contribution from mineral fertilizers and pig slurry (PS) at the rate 68 t ha^−1^ is indicated in brackets.

**Table 2:** Basic physical and chemical properties of soil—variability between experimental plots in 2020–2021.

Parameters	Soil Depth (m)					
	0.0–0.3			0.3–0.6		
	Min–Max	Mean	CV ^1^	Min–Max	Mean	CV
Particle-size distribution						
^2^ Sand, %	14–15	14.3	3.04	13–14	13.8	3.15
Coarse silt, %	23–25	24.5	3.53	22–24	23.0	3.07
Fine silt, %	32–34	33.0	2.14	30–32	31.4	2.75
Clay, %	26–31	28.3	6.32	31–33	31.8	2.61
Chemical properties						
^3^ pH	4.62–6.55	5.25	7.67	4.83–6.98	5.68	10.2
TSC, g kg^−1^	10.9–15.7	14.0	6.48	7.3–17.7	9.93	21.5
TN, g kg^−1^	0.87–1.51	1.29	8.12	0.76–1.35	0.94	13.2
C:N	8.8–15.0	10.9	7.46	8.4–17.6	10.6	19.0
CEC, mM kg^−1^	58.8–101.3	71.3	12.8	54.7–126.0	87.3	18.7
BS, %	57.7–82.9	71.3	7.54	67.0–86.4	77.4	4.90
M3P	14.9–195.7	114.5	42.0	7.3–123.0	52.4	44.3
M3K	132.0–268.0	201.9	15.9	120.6–245.8	181.5	15.2
M3Ca	1177–2786	1731	18.9	1679–4987	2580	27.6
M3Mg	128.0–268.6	196.3	17.0	132.1–298.9	232.2	12.6

^1^ CV—coefficient of variation, in %; ^2^ particle size (sand—0.05–2.0 mm; coarse silt—0.02–0.05 mm; fine silt—0.02–0.05 mm, and clay ≤ 0.002 mm) distribution was analyzed using the aerometric method according to Casagrande, in Prószyński’s modification; ^3^ methods: pH—soil reaction in 1 M KCl (1:2.5, *w*/*v*); TSC—total soil carbon (elemental analysis); TN—total nitrogen content (Kjeldahl method); C:N—TSC:TN; CEC—cation exchange capacity (basic cations in 1 M NH_4_OAc, 1:10, *w*/*v*; and acidic cations in 1 M KCl, 1:2.5, *w*/*v*); BS—CEC saturation with basic cations; M3P, M3K, M3Ca, and M3Mg—Mehlich 3 method.

**Table 3:** The effect of long-term use of pig slurry (PS) and NPK mineral fertilizers on total uptake of macronutrients by winter wheat and sugar beet (mean with 2 × SEM).

Treatment	N_t_	P_t_	K_t_	Ca_t_	Mg_t_
	kg ha^−1^				
Winter wheat (2020)					
N_0_P_0_K_0_	107.4 ± 2.9 ^f^	21.5 ± 1.4 ^b^	56.7 ± 5.3 ^b^	11.5 ± 0.3 ^e^	8.5 ± 0.2 ^e^
N_1_P_1_K_1_	145.2 ± 3.0 ^e^	31.4 ± 3.0 ^a^	81.7 ± 8.1 ^ab^	15.9 ± 0.5 ^cd^	11.6 ± 0.2 ^cd^
N_3_P_2_K_2_	162.0 ± 2.9 ^d^	31.7 ± 2.7 ^a^	85.0 ± 8.1 ^a^	15.9 ± 0.3 ^cd^	10.9 ± 0.2 ^d^
N_4_P_2_K_2_	191.3 ± 4.1 ^bc^	33.7 ± 2.3 ^a^	93.2 ± 3.6 ^a^	18.6 ± 0.5 ^b^	13.0 ± 0.3 ^b^
PS+N_0_P_0_K_0_	159.7 ± 3.4 ^de^	29.9 ± 1.1 ^ab^	77.3 ± 5.4 ^ab^	14.9 ± 0.5 ^d^	12.0 ± 0.3 ^bcd^
PS+N_1_P_1_K_1_	180.2 ± 2.0 ^c^	33.7 ± 1.2 ^a^	81.9 ± 5.7 ^a^	16.0 ± 0.2 ^cd^	12.7 ± 0.1 ^bc^
PS+N_3_P_2_K_2_	200.8 ± 3.7 ^b^	36.2 ± 1.0 ^a^	87.7 ± 5.3 ^a^	17.1 ± 0.3 ^bc^	15.0 ± 0.3 ^a^
PS+N_4_P_2_K_2_	217.9 ± 4.0 ^a^	36.3 ± 1.4 ^a^	89.1 ± 4.9 ^a^	23.4 ± 0.3 ^a^	14.3 ± 0.3 ^a^
ANOVA results					
*F*_7,24_	***	***	**	***	***
Sugar beet (2021)					
N_0_P_0_K_0_	99.7 ± 1.8 ^e^	20.0 ± 0.4 ^e^	149.3 ± 2.8 ^e^	53.8 ± 1.0 ^c^	39.1 ± 0.8 ^e^
N_1_P_1_K_1_	143.2 ± 4.0 ^d^	29.7 ± 0.6 ^d^	216.8 ± 5.6 ^d^	72.2 ± 2.6 ^a^	48.6 ± 1.1 ^d^
N_3_P_2_K_2_	174.0 ± 1.5 ^c^	39.8 ± 0.4 ^b^	275.2 ± 2.6 ^b^	75.0 ± 0.9 ^a^	59.7 ± 0.5 ^c^
N_4_P_2_K_2_	182.1 ± 4.3 ^c^	39.2 ± 0.9 ^b^	292.8 ± 7.0 ^ab^	74.2 ± 1.9 ^a^	59.9 ± 1.4 ^bc^
PS+N_0_P_0_K_0_	145.1 ± 3.9 ^d^	35.7 ± 0.8 ^c^	210.2 ± 5.4 ^d^	73.4 ± 2.5 ^a^	55.4 ± 1.5 ^c^
PS+N_1_P_1_K_1_	180.6 ± 1.1 ^c^	41.1 ± 0.3 ^b^	254.0 ± 1.6 ^c^	74.2 ± 0.4 ^a^	57.0 ± 0.4 ^bc^
PS+N_3_P_2_K_2_	203.3 ± 2.4 ^b^	41.2 ± 0.5 ^b^	281.8 ± 3.3 ^ab^	64.8 ± 0.9 ^b^	60.9 ± 0.7 ^ab^
PS+N_4_P_2_K_2_	274.9 ± 3.2 ^a^	46.3 ± 0.6 ^a^	296.7 ± 3.4 ^a^	59.6 ± 0.7 ^bc^	64.6 ± 0.8 ^a^
ANOVA results					
*F*_7,24_	***	***	***	***	***

***, ** significant at *p* ≤ 0.001, *p* ≤ 0.01, respectively. The same letters indicate a lack of significant difference between the fertilized treatments (HSD test, *p* ≤ 0.05).

**Table 4:** The effect of long-term use of pig slurry (PS) and NPK mineral fertilizers on harvest index (HI) of macronutrients of winter wheat and sugar beet (mean with 2 × SEM).

Treatment	NHI	PHI	KHI	CaHI	MgHI
	%				
Winter wheat (2020)					
N_0_P_0_K_0_	91.4 ± 0.04 ^a^	82.8 ± 0.5	35.3 ± 3.3	26.2 ± 0.1 ^a^	66.6 ± 3.3
N_1_P_1_K_1_	90.3 ± 0.37 ^bc^	80.4 ± 1.5	35.4 ± 3.5	17.3 ± 0.6 ^c^	73.1 ± 3.7
N_3_P_2_K_2_	90.4 ± 0.05 ^bc^	81.3 ± 3.0	35.2 ± 2.9	17.6 ± 0.1 ^c^	66.5 ± 3.0
N_4_P_2_K_2_	90.2 ± 0.32 ^c^	83.3 ± 1.2	33.7 ± 1.7	17.5 ± 0.5 ^c^	75.4 ± 1.8
PS+N_0_P_0_K_0_	91.7 ± 0.27 ^a^	83.7 ± 0.6	37.5 ± 2.1	20.5 ± 0.6 ^b^	73.5 ± 2.0
PS+N_1_P_1_K_1_	91.2 ± 0.01 ^ab^	82.8 ± 0.8	37.6 ± 2.4	16.6 ± 0.1 ^c^	73.9 ± 3.8
PS+N_3_P_2_K_2_	90.4 ± 0.02 ^bc^	82.2 ± 1.6	37.7 ± 3.0	14.6 ± 0.1 ^d^	79.7 ± 1.9
PS+N_4_P_2_K_2_	88.2 ± 0.12 ^d^	85.1 ± 0.8	39.8 ± 4.0	19.8 ± 0.2 ^b^	79.1 ± 4.6
ANOVA results					
*F*_7,24_	***	n.s.	n.s.	***	n.s.
Sugar beet (2021)					
N_0_P_0_K_0_	58.4 ± 1.6 ^ab^	73.5 ± 1.3 ^a^	69.8 ± 1.4 ^a^	61.5 ± 1.6 ^a^	68.1 ± 4.3 ^a^
N_1_P_1_K_1_	50.6 ± 2.1 ^cd^	65.6 ± 1.9 ^cd^	53.4 ± 2.1 ^b^	38.2 ± 2.0 ^bcd^	55.3 ± 2.6 ^b^
N_3_P_2_K_2_	55.5 ± 1.4 ^abc^	66.8 ± 1.3 ^c^	49.8 ± 1.5 ^b^	40.8 ± 1.4 ^bc^	57.7 ± 2.1 ^ab^
N_4_P_2_K_2_	53.8 ± 0.9 ^bc^	66.6 ± 0.8 ^cd^	53.0 ± 0.9 ^b^	44.1 ± 0.9 ^b^	58.4 ± 2.7 ^ab^
PS+N_0_P_0_K_0_	61.2 ± 0.9 ^a^	72.3 ± 0.8 ^ab^	65.3 ± 0.9 ^a^	39.6 ± 0.9 ^bcd^	61.2 ± 2.3 ^ab^
PS+N_1_P_1_K_1_	54.3 ± 0.4 ^bc^	69.5 ± 0.3 ^bc^	54.7 ± 0.4 ^b^	36.3 ± 0.4 ^cd^	56.6 ± 1.5 ^ab^
PS+N_3_P_2_K_2_	53.4 ± 1.0 ^bc^	67.0 ± 0.9 ^bc^	53.4 ± 1.0 ^b^	34.8 ± 0.9 ^d^	53.7 ± 0.7 ^b^
PS+N_4_P_2_K_2_	47.0 ± 1.3 ^d^	61.2 ± 1.3 ^d^	52.2 ± 1.2 ^b^	41.9 ± 1.3 ^bc^	61.3 ± 2.4 ^ab^
ANOVA results					
*F*_7,24_	***	***	***	***	*

***, * significant at *p* ≤ 0.001, *p ≤* 0.05, respectively; n.s.—not significant. The same letters indicate a lack of significant difference between the fertilized treatments (HSD test, *p* ≤ 0.05).

**Table 5:** Content of mineral nitrogen forms in soil and their sum (kg ha^−1^) in early spring before the application of mineral nitrogen fertilizer (mean with 2 × SEM).

Treatments	Soil Depth/Form of N						
	0.0–0.3 m			0.3–0.6 m			0.0–0.6 m
	NH_4_-N	NO_3_-N	N_min_	NH_4_-N	NO_3_-N	N_min_	Total N_min_
Spring 2020							
N_0_P_0_K_0_	2.91 ± 0.26	35.6 ± 3.1	38.5 ± 3.2	2.60 ± 0.28	46.5 ± 7.8	49.1 ± 7.9	87.6 ± 10.1
N_1_P_1_K_1_	2.42 ± 0.24	41.3 ± 9.1	43.7 ± 9.3	3.57 ± 0.22	51.3 ± 9.4	54.8 ± 9.2	98.6 ± 18.5
N_3_P_2_K_2_	2.84 ± 0.21	40.2 ± 6.1	43.0 ± 6.3	2.85 ± 0.19	49.8 ± 6.3	52.7 ± 6.4	95.7 ± 10.1
N_4_P_2_K_2_	2.57 ± 0.15	42.1 ± 3.5	44.7 ± 3.5	2.80 ± 0.37	51.6 ± 4.0	54.4 ± 4.2	99.1 ± 5.8
PS+N_0_P_0_K_0_	2.27 ± 0.07	48.0 ± 1.7	50.3 ± 1.7	2.99 ± 0.15	54.6 ± 5.0	57.5 ± 5.1	107.8 ± 6.8
PS+N_1_P_1_K_1_	2.86 ± 0.37	40.9 ± 3.7	43.8 ± 3.5	3.07 ± 0.66	62.3 ± 4.6	65.4 ± 4.3	109.1 ± 5.0
PS+N_3_P_2_K_2_	2.58 ± 0.34	46.1 ± 2.4	48.7 ± 2.3	2.57 ± 0.38	66.9 ± 5.4	69.5 ± 5.2	118.2 ± 3.8
PS+N_4_P_2_K_2_	3.32 ± 0.10	61.0 ± 9.7	64.4 ± 9.6	3.04 ± 0.53	86.6 ± 19.5	89.7 ± 15.5	147.8 ± 23.3
ANOVA results							
*F*_7,24_	n.s	n.s	n.s	n.s	n.s	n.s	n.s
Spring 2021							
N_0_P_0_K_0_	2.45 ± 0.73	17.7 ± 3.2 ^c^	20.1 ± 3.1 ^d^	5.23 ± 0.75	16.4 ± 1.9 ^c^	21.6 ± 2.7 ^c^	41.8 ± 4.7 ^c^
N_1_P_1_K_1_	3.24 ± 1.01	24.7 ± 2.7 ^abc^	27.9 ± 3.2 ^bcd^	5.07 ± 0.40	21.6 ± 1.6 ^bc^	26.6 ± 1.4 ^bc^	54.5 ± 2.1 ^bc^
N_3_P_2_K_2_	4.36 ± 0.17	25.7 ± 2.4 ^abc^	30.1 ± 2.2 ^abcd^	5.45 ± 0.30	24.3 ± 3.6 ^bc^	29.8 ± 3.3 ^bc^	59.8 ± 5.2 ^bc^
N_4_P_2_K_2_	4.65 ± 0.44	22.4 ± 2.0 ^bc^	27.0 ± 1.7 ^cd^	5.02 ± 0.40	28.4 ± 2.0 ^bc^	33.4 ± 1.9 ^bc^	60.4 ± 1.9 ^bc^
PS+N_0_P_0_K_0_	4.90 ± 0.65	27.8 ± 2.1 ^abc^	32.7 ± 1.8 ^abc^	6.13 ± 0.31	49.0 ± 12.5 ^abc^	55.2 ± 12.2 ^abc^	87.9 ± 11.1 ^ab^
PS+N_1_P_1_K_1_	4.85 ± 0.74	30.2 ± 2.3 ^ab^	35.0 ± 2.7 ^abc^	5.41 ± 0.44	48.8 ± 6.7 ^abc^	54.2 ± 6.7 ^abc^	89.3 ± 4.9 ^ab^
PS+N_3_P_2_K_2_	4.19 ± 1.54	35.4 ± 4.3 ^a^	39.6 ± 3.6 ^ab^	5.52 ± 0.23	57.9 ± 7.4 ^ab^	63.4 ± 7.5 ^ab^	103.0 ± 7.6 ^a^
PS+N_4_P_2_K_2_	5.40 ± 0.57	35.3 ± 0.7 ^a^	40.7 ± 1.2 ^a^	4.91 ± 0.32	66.8 ± 16.1 ^a^	71.7 ± 16.4 ^a^	112.4 ± 17.0 ^a^
ANOVA results							
*F*_7,24_	n.s	***	***	n.s	***	***	***

***, significant at *p* ≤ 0.001; n.s.—not significant. The same letters indicate a lack of significant difference between the fertilized treatments (HSD test, *p* ≤ 0.05).

**Table 6:** Correlation matrix—winter wheat grain yield (GY), straw yield (SY), sugar beet taproot yield (TY), leaf yield (LY), total macronutrient accumulation in plants, and macronutrient contents in two soil depths (*n* = 8).

Features	GY/TY	SY/LY	N_t_	P_t_	K_t_	Ca_t_	Mg_t_
Winter wheat (2020)							
Soil depth: 0.0–0.3 m							
N_min_	0.64	0.53	0.74 *	0.61	0.48	0.85 **	0.68
M3P	0.90 **	0.93 **	0.93 **	0.94 ***	0.80 *	0.76 *	0.91 **
M3K	0.57	0.57	0.59	0.58	0.33	0.35	0.72 *
M3Ca	−0.66	−0.67	−0.87 **	−0.69	−0.58	−0.75 *	−0.79 *
M3Mg	−0.21	−0.38	−0.33	−0.41	−0.61	−0.40	−0.18
Soil depth: 0.3–0.6 m							
N_min_	0.69	0.64	0.81 *	0.71 *	0.48	0.81 *	0.79 *
M3P	0.86 **	0.88 **	0.89 **	0.89 **	0.72 *	0.69	0.90 **
M3K	0.76 *	0.79 *	0.76 *	0.80 *	0.60	0.61	0.83 *
M3Ca	−0.58	−0.47	−0.60	−0.42	−0.29	−0.32	−0.52
M3Mg	−0.22	−0.33	−0.21	−0.27	−0.54	−0.10	−0.06
Sugar beet 2021							
Soil depth: 0.0–0.3 m							
N_min_	0.85 **	0.80 *	0.84 **	0.86 **	0.69	0.09	0.81 *
M3P	0.79 *	0.83 *	0.85 **	0.85 **	0.77 *	0.02	0.80 *
M3K	0.74 *	0.88 **	0.82 *	0.81 *	0.88 **	0.14	0.80 *
M3Ca	−0.65	−0.58	−0.71 *	−0.71 *	−0.57	0.19	−0.68
M3Mg	−0.16	−0.43	−0.34	−0.27	−0.57	−0.10	−0.32
Soil depth: 0.3–0.6 m							
N_min_	0.75 *	0.62	0.77 *	0.75 *	0.52	−0.09	0.70
M3P	0.78 *	0.81 *	0.91 **	0.80 *	0.84 **	−0.02	0.82 *
M3K	0.73 *	0.78 *	0.77 *	0.72 *	0.82 *	0.09	0.75 *
M3Ca	−0.70	−0.57	−0.45	−0.61	−0.62	−0.37	−0.68
M3Mg	−0.19	−0.32	−0.09	−0.20	−0.47	−0.50	−0.28

***, **, * significant at *p* ≤ 0.001, *p* ≤ 0.01, *p* ≤ 0.05, respectively.

**Table 7:** Nitrogen use efficiency (NUE) indices as a result of long-term application of pig slurry (PS) and mineral fertilizers (NPKs).

Treatment	PFP kg kg^−1^	AE kg kg^−1^	PNB kg kg^−1^	NUtE kg kg^−1^	NUEs kg kg^−1^	PFP_in_ kg kg^−1^	NUpE kg ha^−1^	NUEb kg ha^−1^
Winter wheat (2020)								
N_0_P_0_K_0_	---	---	---	47.9 ± 0.02 ^b^	104.3 ± 2.8 ^b^	58.6 ± 1.6 ^b^	122.5 ± 3.3 ^b^	---
N_1_P_1_K_1_	175.9 ± 3.8 ^b^	47.4 ± 3.8 ^bc^	2.68 ± 0.07 ^c^	48.4 ± 0.20 ^a^	92.3 ± 2.0 ^ac^	50.8 ± 1.1 ^c^	104.8 ± 2.1 ^ef^	3.28 ± 0.07 ^b^
N_3_P_2_K_2_	133.1 ± 2.4 ^d^	39.7 ± 2.4 ^cd^	2.64 ± 0.05 ^c^	45.2 ± 0.02 ^c^	87.9 ± 1.6 ^c^	48.6 ± 0.9 ^cd^	107.5 ± 1.9 ^cde^	2.66 ± 0.05 ^c^
N_4_P_2_K_2_	103.2 ± 2.3 ^e^	34.7 ± 2.3 ^d^	2.16 ± 0.04 ^d^	40.5 ± 0.15 ^f^	80.9 ± 1.7 ^d^	44.5 ± 1.0 ^e^	109.8 ± 2.3 ^de^	2.30 ± 0.05 ^d^
PS+N_0_P_0_K_0_	---		---	48.2 ± 0.14 ^ab^	124.4 ± 3.2 ^a^	71.5 ± 1.5 ^a^	148.2 ± 3.2 ^a^	---
PS+N_1_P_1_K_1_	196.3 ± 2.2 ^a^	67.9 ± 2.1 ^a^	3.99 ± 0.09 ^a^	43.6 ± 0.01 ^d^	95.4 ± 1.0 ^bc^	52.7 ± 0.6 ^c^	120.8 ± 1.3 ^bc^	4.11 ± 0.04 ^a^
PS+N_3_P_2_K_2_	151.7 ± 2.8 ^c^	58.3 ± 2.8 ^ab^	3.28 ± 0.04 ^b^	41.6 ± 0.01 ^e^	87.3 ± 1.6 ^cd^	48.2 ± 0.9 ^cd^	115.9 ± 2.2 ^bcd^	3.30 ± 0.06 ^b^
PS+N_4_P_2_K_2_	110.1 ± 2.1 ^e^	41.6 ± 2.1 ^cd^	2.68 ± 0.05 ^c^	37.9 ± 0.05 ^g^	64.4 ± 1.1 ^e^	36.1 ± 0.7 ^d^	95.2 ± 1.8 ^f^	2.56 ± 0.05 ^c^
Sugar beet (2021)								
N_0_P_0_K_0_	---	---	---	614.9 ± 17.1 ^a^	1963.3 ± 40.3 ^a^	1467.9 ± 48.4 ^a^	238.7 ± 4.2 ^a^	---
N_1_P_1_K_1_	903.3 ± 26.9 ^a^	137.1 ± 26.9 ^b^	1.25 ± 0.02 ^b^	505.7 ± 21.2 ^bc^	816.5 ± 16.9 ^c^	537.1 ± 16.0 ^c^	106.5 ± 3.0 ^c^	0.90 ± 0.03 ^b^
N_3_P_2_K_2_	503.3 ± 13.6 ^b^	120.1 ± 13.6 ^b^	0.90 ± 0.02 ^c^	462.8 ± 12.0 ^cd^	584.1 ± 5.5 ^d^	366.3 ± 9.9 ^d^	79.1 ± 0.7 ^de^	0.60 ± 0.02 ^c^
N_4_P_2_K_2_	408.2 ± 9.1 ^c^	101.7 ± 9.1 ^b^	0.87 ± 0.01 ^c^	448.6 ± 7.5 ^d^	492.2 ± 11.1 ^de^	313.5 ± 7.0 ^d^	69.9 ± 1.7 ^e^	0.49 ± 0.01 ^d^
PS+N_0_P_0_K_0_	906.0 ± 16.0 ^a^	217.2 ± 16.0 ^a^	2.05 ± 0.05 ^a^	556.4 ± 8.2 ^b^	1352.1 ± 33.5 ^b^	917.3 ± 16.2 ^b^	165.0 ± 4.4 ^b^	1.00 ± 0.02 ^a^
PS+N_1_P_1_K_1_	483.4 ± 5.5 ^b^	120.7 ± 5.5 ^b^	0.86 ± 0.02 ^c^	452.3 ± 3.4 ^cd^	750.7 ± 5.3 ^c^	482.6 ± 5.5 ^c^	106.7 ± 0.7 ^c^	0.58 ± 0.01 ^c^
PS+N_3_P_2_K_2_	335.6 ± 7.1 ^d^	89.4 ± 7.1 ^b^	0.73 ± 0.01 ^d^	411.0 ± 7.6 ^d^	507.0 ± 6.1 ^de^	317.7 ± 6.7 ^d^	77.3 ± 0.9 ^de^	0.44 ± 0.01 ^d^
PS+N_4_P_2_K_2_	297.8 ± 8.9 ^d^	85.7 ± 8.9 ^b^	0.70 ± 0.01 ^d^	313.1 ± 9.0 ^e^	438.2 ± 6.0 ^e^	275.5 ± 8.2 ^d^	88.0 ± 1.0 ^d^	0.45 ± 0.01 ^d^

The same letters indicate a lack of significant difference between the fertilized treatments (HSD test, *p* ≤ 0.05). Key: PFP—partial factor productivity of N in fertilizers (N_f_); AE—agronomic efficiency; PNB—partial N fertilizers balance; NUtE—N utilization efficiency; NUEs—biomass productivity of N_in_ (=N_min_ + N_f_); PFP_in_—partial factor productivity of N_in_; NUpE—N uptake efficiency; NUEb—N input/output balance.

## Data Availability

Dataset available on request from the authors.

## Supplementary Materials

The following supporting information can be downloaded at: https://www.mdpi.com/article/10.3390/life15111779/s1, Figure S1: Winter wheat grain yield (GY) as a function of plant-available P (a) and K (b) content in two soil depths; Figure S2: Sugar beet taproot yield (TY) as a function of plant-available P (a) and K (b) content in two soil depths; Table S1: The effect of fertilization variants on the quality of the crop and total protein content in winter wheat grain and sucrose content in sugar beet roots.
