# Supplementary material for: Response of Wheat and Sugar Beet to Different Mineral–Organic Fertilization in a Long-Term Experiment
Source: Life (Basel). 2025 Nov 20;15(11):1779. doi: 10.3390/life15111779 (PMC12654082; doi:10.3390/life15111779)
Supplement: Supplementary file 1 [file life-15-01779-s001.zip › life-3949679-supplementary.pdf]

Supplementary material

Response of Wheat and Sugar Beet to Different Mineral-Organic Fertilization in a Long-Term Experiment

Przemysław Barłóg[1]*, Lukáš Hlisnikovský[2], Remigiusz Łukowiak[1], Ladislav Menšík[2] and Eva Kunzová[2]

[1] Department of Agricultural Chemistry and Environmental Biogeochemistry, Poznan University of Life Sciences, Wojska Polskiego 71F, 60-625 Poznan, Poland; przemyslaw.barlog@up.poznan.pl (P.B.); remigiusz.lukowiak@up.poznan.pl (R.Ł.).

[2] Department of Nutrition Management, Crop Research Institute, Drnovská 507, CZ-161 01 Prague 6, Ruzyně, the Czech Republic; lukas.hlisnikovsky@carc.cz (L.H.); ladislav.mensik@carc.cz (L.M.), eva.kunzova@carc.cz (E.K.)

* Correspondence: przemyslaw.barlog@up.poznan.pl

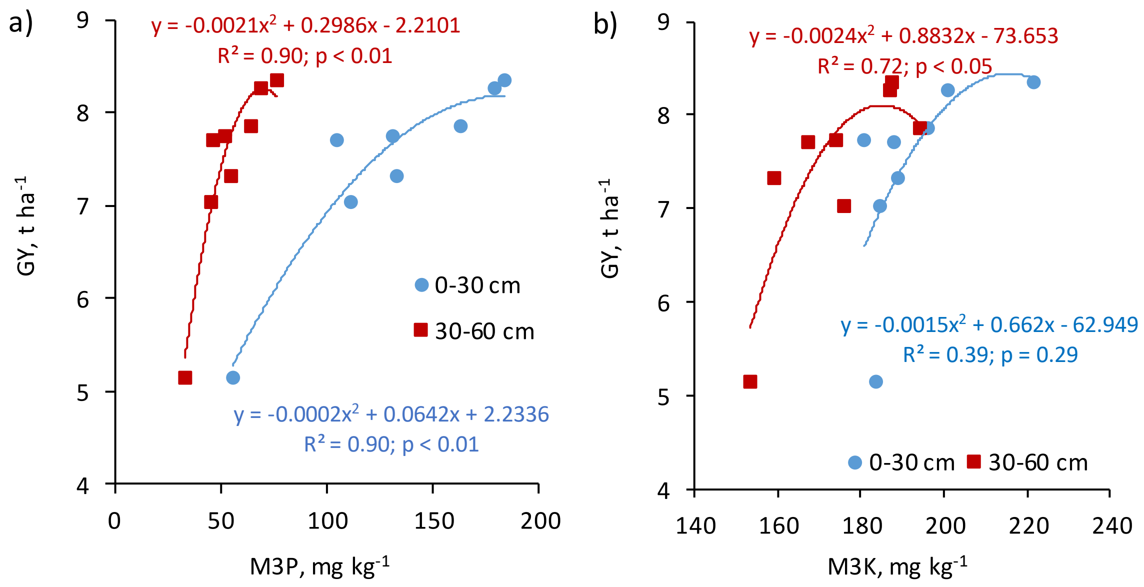

Figure S1. Winter wheat grain yield (GY) as a function of plant-available P (a) and K (b) content in two soil depths.

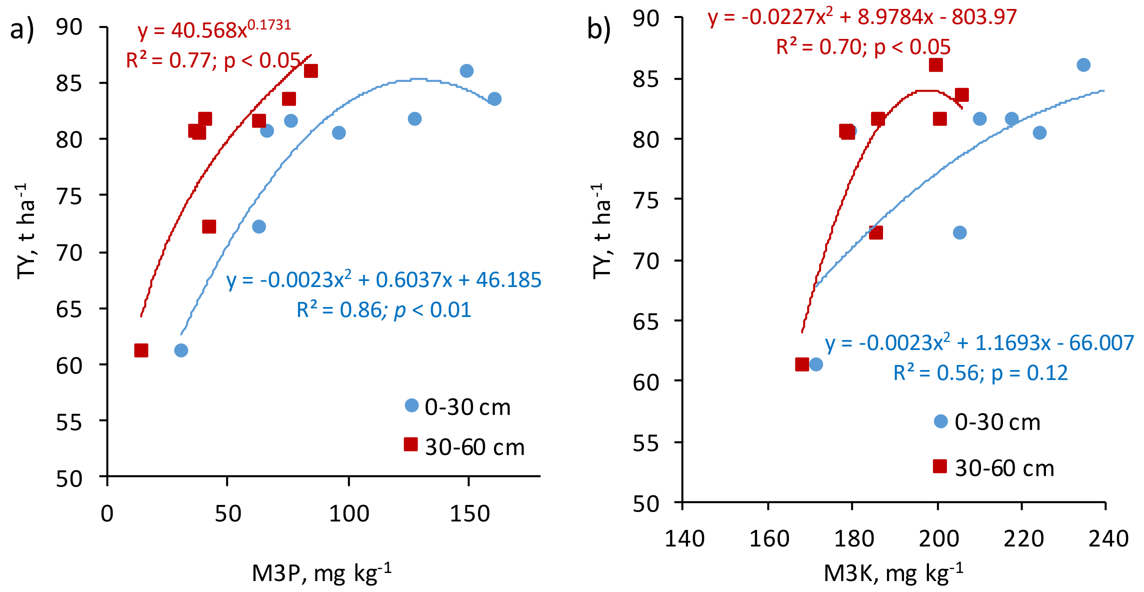

Figure S2. Sugar beet taproot yield (TY) as a function of plant-available P (a) and K (b) content in two soil depths.

Table S1. The effect of fertilization variants on the quality of the crop: total protein content in winter wheat grain and sucrose content in sugar beet roots.

**Treatment**	**Winter wheat % of crude protein**	**Sugar beet % of sucrose in taproots**
$N_0P_0K_0$	11.9	18.8
$N_1P_1K_1$	11.6	19.5
$N_3P_2K_2$	12.5	19.4
$N_4P_2K_2$	13.9	19.6
PS+$N_0P_0K_0$	11.9	19.4
PS+$N_1P_1K_1$	13.1	19.2
PS+$N_3P_2K_2$	13.6	19.0
PS+$N_4P_2K_2$	14.6	18.6

where PS denotes for pig slurry application and 1,2,3,4 different rates of mineral NPK fertilizers
